# Assessment of Directly Observed Therapy (DOT) following tuberculosis regimen change in Addis Ababa, Ethiopia: a qualitative study

**DOI:** 10.1186/s12879-015-1142-2

**Published:** 2015-09-30

**Authors:** Daniel Fiseha, Meaza Demissie

**Affiliations:** Tuberculosis research advisory committee of the Federal Ministry of Health, Addis Ababa, Ethiopia; KNCV Tuberculosis Foundation, Challenge TB, Country Office, Addis Ababa, Ethiopia; Addis Continental Institute of Public Health, Addis Ababa, Ethiopia

**Keywords:** Tuberculosis, Qualitative research, Ethiopia

## Abstract

**Background:**

Tuberculosis remains a major public health problem in Ethiopia. In 2010 the TB treatment regimen was shortened from 8 to 6-months treatment. With this new regimen, the full course of treatment should be taken under Directly Observed Therapy (DOT) unlike the 8-month regimen where TB patients were only observed during the intensive phase, this has not been tried before and may be difficult to implement. Therefore this study aimed to investigate the experiences from both TB patients and health care providers’ perspective of implementing DOT for the full course of TB treatment.

**Methods:**

Qualitative study consisted of 11 in-depth interviews and 4 Focus Group Discussions (FDGs) were conducted between March and April, 2014. Overall, 18 TB patients and 16 HCPs were involved from three selected public health facilities (2 Health Centers and 1 Hospital) in Addis Ababa, Ethiopia. Qualitative data analysis software (Open Code Version 3.5) was employed to identify the key issues from these interviews through coding, categorization and grouping into emergent themes.

**Results:**

Participants reported that making a daily visit to health facilities for DOT was difficult due to the distance of the facilities from their residences, lack of or high transportation cost and had undesired implications on their work and social lives. TB patients had to overcome many challenges to comply with TB treatment on a daily basis. HCPs also indicated the difficulties of implementing facility based daily DOT mainly due the implication it had on their TB patients and stated DOT had not always been implemented for the full course as recommended. HCPs also shared deep concern regarding the risk of acquiring multiple drug resistant TB.

**Conclusion:**

This study indicated there are several challenges associated with facility based daily DOT as a method of TB treatment supervision in public health facilities in Addis Ababa. This may be indicative of the situation in other health facilities in Addis Ababa as well as elsewhere in the country. Hence the TB control program has to explore how best to improve TB treatment delivery options to ensure adequate treatment. A more patient-centered approach could be strengthened by further decentralizing the DOT to the community level in order to ensure adherence of patients to their TB treatment.

## Background

Tuberculosis (TB) remains a major global health problem. About a third of the world’s population is estimated to be infected with Mycobacterium tuberculosis. Ethiopia stands 7th among the 22 High-Burden Countries (HBCs) and these countries account for about 80 % of the world's TB cases [1, 2]. In Addis Ababa, the principal city of Ethiopia, TB is also a major public health concern [3]. The National Tuberculosis Program (NTP) efforts to control TB in Ethiopia began in the early 1960s through the adoption and implementation of different internationally recommended TB control strategies. Ethiopia has been implementing the World Health Organization (WHO) recommended TB control strategy known as Directly Observed Treatment Short- Course (DOTS) since 1991 and also adopted the WHO Stop TB Strategy since 2006 [4].

Since the year 2004 WHO recommended all countries and settings to change their TB treatment from 8-month to 6-month with the shift of the continuation phase of the anti-TB regimen from Ethambutol and Isoniazid (EH) based regimen to Rifampicin and Isoniazid (RH) based regimen. Hence, the full course of treatment is expected to be directly observed [5, 6]. This recommendation was based on the trial of the International Union Against Tuberculosis and Lung Disease (The Union) demonstrating increased efficacy of the 6-month regimen and was shown to be clinically superior to the 8-month regimen [7] also the 8-month regimen was associated with more relapses and deaths than the 6-month regimen [8]. In addition, shorter regimens for drug-susceptible TB may increase adherence, reduce default, attract more TB patients, and bring higher effective cure rates and fewer new cases of multiple drug-resistant TB (MDR-TB). Therefore, the identification of lengthy regimens as a problem and treatment shortening as a global goal have been formalized by the Stop TB Partnership in the Global Plan 2006–2015 [2]. However of the 22 HBCs Ethiopia and Nigeria delayed the change from the 8- month to the 6-month regimen and the main reasons reported were primarily due to a perceived lack of strong DOT, fear of poor adherence and thus concern about increasing Rifampicin resistance [9].

But since September 2010, the Ethiopian Federal Ministry of Health, decided to implement the regimen change across the country in a phased manner and the regimen change was initially piloted in selected facilities found in three urban regions of the country which was followed by full scale-up into all the public health facilities in the country with strong consideration for implementation of DOT for the whole six month duration of patients treatment [4, 10]. DOT that is watching the patient swallow every scheduled dose, in the right doses and at the right intervals, is the most effective method to promote treatment adherence and gives individual TB patients the best chance of cure and also avoids the emergence of drug resistance [11]. With this new regimen, the full course of treatment should be taken under DOT to make sure patients are taking all the prescribed drugs. This means patients should be observed while taking their medications on daily basis by TB Treatment Supporters (TTS) for the full six months of treatment duration, unlike the previous regimen where TB patients were only observed during the intensive phase during the first two months of their treatment and this has not been tried before and might have some consequences as it may be difficult for all TB patients to be observed while taking each dose of their medications every day for the total duration of their treatment. Therefore this study aimed to explore the experiences from both patients and health care providers (HCPs) of implementing DOT for the full course of TB treatment in selected public health facilities found in Addis Ababa using qualitative methods.

## Methods

### Study setting

The study was conducted in three selected public health facilities (two health centers and one hospital) found in Addis Ababa, the principal city of the Federal Democratic Republic of Ethiopia. Addis Ababa has a projected total population of 3,272,237 (52 % Female and 48 % Male) in the year 2014/15 and the HIV prevalence among adult population in 2011 was 5.2 %. In the city there were a total of 102 public health facilities (88 Health centers and 14 hospitals including federal, police and defense hospitals) providing TB diagnosis and treatment services [3] and in total 24.8 % of all households are considered overcrowded [12].

### Study design

A qualitative study method consisted of 11 in-depth interviews and 4 focus group discussions (FDGs) were conducted and the data collection period was between March and April, 2014.

### Study participants

A total of 34 participants (18 TB patients and 16 HCPs) were involved from three selected public health facilities (2 Health Centers and 1 Hospital) in Addis Ababa, Ethiopia. A total of 11 participants involved in the in-depth interviews until we reached redundancy and the rest 23 in the 4 FGDs divided into two sub groups: TB patients group and HCPs group, categorized by gender. Interviewed TB patients (IP) were those patients who visited the three public health facilities for their TB treatment under DOT during the data collection period and the HCPs were those with long work experience and involvement in the management of TB patients in the respective public health facilities as DOT Providers (DP).

### Data collection and procedure

All the in-depth interviews and FGDs were conducted by the principal investigator and a well trained health officer who served as assistant data collector and was involved in the facilitation of the data collection processes. At the beginning and end of each of the data collection days we had a debriefing meeting where we had an opportunity to discuss any issues related to interviews, these activities had repeated till the end of the data collection. Individual’s patient and DOT provider interviews took place in private area inside the facilities or TB clinics. Prior facility visits were made to explain about the research objectives and to get permission from the heads of the respective facilities to conduct the research and to make arrangements for data collection purpose.

Each Interview was done using a semi-structured questionnaire and an interview guide which was developed by consulting different literatures and national documents [4, 10]. For the patient group questions include “perception and experiences related to DOT”, “Support from family, friends and the community during their TB treatment” and “Health care facilities and interactions with HCPs” and for the health care providers group: “their experiences in providing DOT”, “Main problems related to observation of treatment”, “any suggestion for improvements (DOT)”. These questions were initially developed in English and translated into Amharic (local language).

Each FGD was conducted in a selected room which could accommodate the number of participants. Each session of the FGDs lasted an average of 90 min. A FGDs Interviews questions and guide were used to initiate discussions and to gear the whole session of the discussions towards the topic. All sessions of the in-depth interviews and the FGDs were conducted in Amharic language and were audio-recorded.

### Data analysis data quality assurance

Data analysis was started together with the data collection process. All the audio records of the interviews, the FGDs and the field notes were reviewed on a daily basis. Each of the audio record was repeatedly listened to and transcribed verbatim and translated into English by the principal investigator. All the field notes, transcripts and translations were read repeatedly till the investigator becomes familiar with them. Qualitative data analysis software (Open Code Version 3.5) was employed for the development of coding based on the original terms used by participants and this was followed by identifying the most frequently observed category and the development of emergent themes.

### Ethical considerations

Ethical clearance was obtained from the Institutional Review Board (IRB) of University of Gondar, from Addis Ababa city administration health bureau ethical clearance committee and St Peter TB Specialized hospital ethical clearance board. Prior to each session, a written description of the study aims and risks of participating was provided. Written informed consent to participate was obtained from each participant of the study and to ensure participant’s anonymity and privacy during interviews private areas were used and following the data collection from each study participants’ audio records were kept confidential. Each study subject was identified only by code. Also the collected data were kept secure with the principal investigator.

## Results

A total of thirty-four participants (18 TB patients and 16 health care providers) were involved in both in-depth interviews and four focus groups (Table 1 and 2). The most relevant themes identified from the analysis are listed below and each theme is reported in detail with illustrative quotes from data.

**Table 1:** TB patients participated from public health facilities in Addis Ababa, Ethiopia, March and April /2014

Characteristics	In-depth interview	FGDs
	TB patients (*n* = 7)	TB Patients (*n* = 11)
Gender		
Male	3	6
Female	4	5
Age		
< 25	2	2
26–35	3	6
> 36	2	3
TB treatment facility		
Health Center 1	4	11
Health Center 2	2	
Public Hospital	1	
Treatment durations		
Intensive phase (<2 months)	4	8
Continuation phase (>2 months)	3	3

**Table 2:** HCPs participated from three public health facilities in Addis Ababa, Ethiopia, March and April /2014

Characteristics	In-depth interview	FGDs
	TB patients (*n* = 4)	TB Patients (*n* = 12)
Gender		
Male	3	6
Female	1	6
TB treatment facility		
Health Center 1	1	12
Health Center 2	1	
Public Hospital	2	
Professions		
BSc /Clinical nurse	2	7
Health officers	2	5

1. TB patients regard daily facility visits challenging
2. Most TB patients comply with treatment
3. Family support described as vital to continue treatment
4. DOT in continuations phase was difficult to implement
5. HCPs reported risk of acquiring MDR-TB
6. TB patients regard daily facility visits challenging

Most TB patients reported that coming daily to the TB clinic especially in the first two months of treatment was very difficult and physically demanding and few patients regard it as unnecessary. This was particularly the case for the many patients who were severely sick at the time of initiating treatment. Also majority of the patients often come the clinic on foot every day due to lack of money or due to the high cost of transportation. A female patient described her daily visits experiences to one the health centre as follows:

*“For me coming daily has no benefit because anyone has to live for him or herself. Actually what they (DOT providers) think is to prevent the interruption of taking our medications but there are patients who are bed ridden and coming here daily would be very challenging for them . Myself, I have to come here on foot on daily basis but the road is hilly and exhaustive and I had to take several rests on my way because I’m not yet fully recovered and I was discharged from hospital recently. If I get advices not interrupt my drugs and given to take at home I would be very happy and will take my medication strictly” - (IP 1).*

All the DOT providers interviewed acknowledged that application of strict daily DOT for all TB patients was very challenging to implement due the problems imposed to their TB patients. They describe most TB patients are very poor and less educated with families to support and coming daily has implications on work, income and social life of patients. They also describe, in their daily encounters that most patients usually beg them to give drugs for more days to take at home.

*“TB patients come on daily basis to our facility to take their medications but sometimes very challenging for them to come daily. We encountered with TB patients* who *are very poor and who support themselves and their families by working as a daily laborer, these patients usually complain about coming daily is interfering with their work and earnings and they have nothing to eat unless they work on time. Few of the patients are also working in private organizations and getting permission from their employer to come to the facility for their treatment was difficult and for those reasons it is always difficult for us to implement DOT strictly for all TB patients and we sometime give patients with their drugs to take them at home but we also make them to come in long interval to check for side effects and other problems” - (DP 3)*

2) Most TB patients comply with treatment

The majority of TB patients interviewed said they wanted to be cured and one patient said she feels she will die if she does not take her drug for one day. Most patients also reported that the advice they got from the health care providers (DOT providers) helped them to continue their treatment and most patients described the interaction they had with the DOT provider as positive. Most patients also reported that DOT providers observe them while swallowing their drug, also stated they get important information about TB disease, what foods to eat and about side effects of drugs from their DOT providers. Below is a description of a male TB patient in continuation phase of treatment.

*“I come here daily to take my medications empty stomach and now on my 4th month of treatment, I’m so much grateful to the doctor who is working here in the TB Clinic. You know there was* no one *like me who loves to drink alcohol even if being on treatment for TB but the doctor’s continuous advice helped me to quit drinking until I finish my treatment. In addition I’m taking care of myself and will continue my treatment properly because I accepted the advice “(IP 2)*

Few TB patients expressed their concerns and discomfort regarding the frequent change of the health care providers at the TB clinic at different time during the course of their treatments and they also pointed out they feel unconfident/uncomfortable with some of the health care providers: Below is a 28 years old female patients expressing her feeling regarding encountering of different health care providers.

*“The current DOT provider in the TB clinic is the fourth person in the row I encountered since I started my treatment here. I’m not against and I don’t oppose the change of the health care providers in the TB clinic but all of them should be aware of what drugs I’m taking and I think this should be a must but few of them ask what drugs I’m taking and they look confused with the registers and with what drugs to give to me to take them “(Female FGD participant)*

Most health care providers described there is no permanent health care provider assigned to work in the TB clinic rather different health care providers are assigned on rotation and working for a certain time interval . Sometimes a health care provider who is not formally trained on TB management may be obliged to work in the TB clinic and this could compromise the quality of the service and the HCPs suggested to the concerned body to provide training on the management of TB for all health care providers working in the respective facilities. Below is how a DOT provider described the importance of assigning a permanent staff at the TB clinic in his facility.

*“I strongly suggest assigning of full time dedicated and permanent staff in the TB clinic who can consistently offer the TB treatment and also important information and advice to all TB patients during their treatment but this is not happening and there is no full time dedicated TB clinic staff. For instance I myself not working permanently here in this TB clinic rather it is additional responsibility beside working in the OPD and this is a big burden for me and as a human being I may* burn out and loss *concentration and might not treat patients politely and may not offer them the right information and advice even sometime I may committee mistakes even quarrel with patients therefore assigning permanent staff is I think very important” (DP 2*)

3) TB patients feel that the close family support is vital

The majority of TB patients stated they told to and close family members know their TB status, who in turn provided them support and encouragement during treatment. However, few respondents said they often feel stigmatized by few close friends and neighbors because of a lack of understanding about TB. Below a female patient described the good support from her close families and the discomfort regarding her interaction with old friends and what another male TB patient told:

*“My family all of them including my mother and sisters are supportive when I was* admitted *at “Samba nekersa” (local hospital) they never* get *far away from my side. They were always available at the time of need especially my mom* she *always weak up early in the morning to remind me to* come *here (TB Clinic) I’m really thankful for her support. But regarding neighbors they put a big pressure on me when I say pressure they keep distant from me including my childhood friends with whom I used to hang and play but I understand them all it is because of their fear but as far as I’m taking my drugs after 15 days I cannot transmits TB to them that is what the doctors told me. I also take care not to infect others I always covers my* mouths *and nose whenever I’m coughing “(IP 3).*

*“I told to my close friends about my TB disease and other people around my neighborhood also know about my conditions but nobody comes to my home to ask and to talk to me except very few close friends who used to encourage me saying ‘Egiziabiher yimarih’(May God cure you) when they met me outside . I don’t complain much and I accepted the situations and I always pray crying and tells to my God “(IP 4)*

Most of the health care providers interviewed also expressed TB patients are stigmatize due to lack of knowledge and awareness about TB by the community. The health care provider recommended TB related education should be continuously done by different means and Medias including through radio, Television, posters and leaflets. One health care provider participated in one of the focus group underlined the importance of giving educations to the community including testimonials by cured TB patients.

*“I myself don’t want to use the bus serving around this facility because MDR-TB patients are using it to travel to the hospital to get their treatments. The bus is also known by the nick name MDR- bus and if you see this bus it is always full of people and the passengers don’t want to open any of the windows due to the fear of ‘bird’ (Cold air) and if you are inside the bus and you wanted to open the windows you cannot open any of them because many of the windows are deactivated to be un open able for the purpose of posting an advertisements” (Male HCPs FGD participant).*

4) No DOT in continuation phase

The national TB program introduced the RH containing regimen in the continuation phase of TB treatment although recommended treatment procedure is DOT for the whole course and duration of treatment including in the continuation phase but most of the health care providers reported DOT is not implemented as recommended.

Most stated that they are aware of and were trained on the six month RH containing treatment regimen which should be provided under strict observation of all TB patients for the whole duration of their treatments but this was very difficult to implement mainly due to problems and challenges impose on their TB patients. DOT could have been done at the community level but there was no established mechanism to work with the Health Extension Workers (HEWs) or other community members who can serve as TB TTS and observe patients while taking their medications in their home. Below is how a DOT provider and a female health professional participant of focus groups describe the importance of DOT and the challenges related to implementing supervised treatment for six months.

*“I suggest DOT should be implemented for the whole course of six months for all TB patients but* there *are several problems to do that. For example few TB patients are government* employee *which is very difficult for them to get permission or sick leave to come here daily for their treatments and there are also few private employed patients who will not be able to get any permission and even they may be fired if known to have TB disease by their employer. For those reason it is a usual encounter that TB patients always put big pressure on us to give them their medication to taking them medication at their home. These are what patients* usually *tell and this is the idea they present often and it is difficult.” (DP 1*).

*“DOT for TB treatment is always a controversy for me because TB* patients *always argue with us not to come every day particularly those patients who are students they always beg us to give them* drugs *for more days to take at home and I know long time ago TB was recognized as dangerous disease and the DOT program was introduced as means of treatment delivery for TB patients but some patients pressurized us mentioning they know about the disease seriousness and the importance of taking their medication and if given they can take it at home . Few also tell us they encounter with big social problem and beg us to give them drugs for more days and we give them. I know from the point of preventing the generation of MDR-TB DOT is very important but it is difficult from patients perspectives” (Female FGD participant)*

5) Health care providers reported risk of MDR-TB

The risk of TB/ MDR-TB transmission from patients to healthcare workers was a major issue raised by all the participants. To the providers, TB infection especially MDR-TB represents a serious concern for most. The two health centers in study are MDR-TB treatment follow-up center (TFC) and the hospital is an MDR-TB treatment initiating center (TIC) and the HCPs stated they felt and are concerned that this might put them at increased risk of acquiring TB/MDR-TB from patients, though there was no health care provider reported with MDR-TB diagnosed. Below is how one health professional working in one of the health center describes her and another colleague’s concern about work at a TB clinic.

*“Safety and protecting the HCPs working here (TB Clinic) is very concerning, as you know* these days *MDR TB is coming into the picture and I don’t perceive much is being done to* protect *the HCPs who are assigned and working the TB clinic. These days it is becoming hard to get a health care provider who is willing to work in a TB clinic because of the risk and fear acquitting TB/MDR-TB and I think this issue must be thoroughly investigated and considered” (Female FGD Participant).*

*“The TB clinic is below the standard, the roof height is short, the door and the windows should be easily open able and in opposite position these all are not in place as you can see. This will definitely expose us to infection. We are working here to help patients and this has to be corrected, imagine yourself working in this room and condition this is very risky and for us this is a big problem “. (DP 3)*

## Discussion

In this study most participants reported that making a daily visit to health facility for DOT was difficult due to the distance of the facilities from their residences, lack of or high transportation cost and had undesired implications on their work and social lives. TB patients need to overcome many of these challenges to comply with TB treatment on a daily basis although most TB patients do comply with treatment because they wanted to be cured and the advices from their DOT providers and close family supports were also important for most TB patients. HCPs also indicated the difficulties of implementing facility based DOT mainly due the implication it had on their TB patients and stated DOT had not always been implemented for the full course as recommended also reported deep concern regarding risk of acquiring MDR-TB while working in the TB clinics.

The national TB program has introduced the 6-month TB treatment regimen containing RH in the continuation phase, this regimen has several benefits, it has a lower rate of treatment failure and relapse and it shortens the total duration TB treatment and could increase patient’s compliance and adherence [2]. But the most important disadvantage of this regimen is the possibility of development of Rifampicin resistant TB in patients with poor adherence and MDR-TB could increase if strict DOT is not followed for the full course of treatment [4, 10].

In this study we found that in all the three public health facilities DOT was not implemented in continuation phase of TB patient’s treatment because of the challenges and difficulties imposed on the TB patients. Daily facility visits for DOT during the intensive phase was not acceptable by most of the TB patients and has several implications on their work, family and social lives and it was unrealistic for the DOT providers to make the whole course of the TB treatment under DOT. The Ethiopian national TB guideline suggest that DOT could be implemented in different settings not only in a health facility but also at patients home and could be done by HEWs or other trained community members who could observe patients while taking their medications down in the community [3, 4]. This approach was found to be more cost effective and efficient in rural Bangladesh as compared to facility based DOT. But in this study DOT was not yet established at the community level and across the three facilities and it seems very important for the national TB program to established a mechanism to make sure DOT is implemented for entire period of TB treatment in all settings otherwise the risk of MDR-TB could potentially exists if adherence to treatment cannot be verify.

For treatment adherence personal characteristics of patients like the knowledge, motivations and perceptions about disease are important determinant factors for adherence to treatment [13]. TB related information, health education, and consultation by the DOT provider for patients could help to increase the knowledge and understanding of the TB patients about their disease condition could motivate them to adhere to their treatment [14]. The support from the family and community is also very important for patients to comply with their treatment. These supports could be financial or psychological. Most of the TB patients in this study comply with treatment despite the several challenges they are facing, because they wanted to be cured this could indicate the importance of individual TB patient understanding, determination and motivation to complete treatment. Also health care providers, families and the community could play a vital role in encouraging and supporting TB patients to comply with treatment strategy to increase the knowledge and awareness of TB patient’s family and the community about TB should be important activity for a TB control program.

Facility based daily DOT was associated with several challenges and with implications on patients in terms of their work, social and family lives but despite those challenges TB patients in this study still wanted to continue with their treatment because they wanted to be cured and the advice,encouragement and support from both the DOT providers and family were also important for continuing of their TB treatments and these findings were similar with a study conducted in Addis Ababa found that despite several challenges a large group of patients still managed to continue treatment, mainly because relatives or community members provided food, encouragement and sometimes money for transport [15] another qualitative study conducted among TB/HIV co-infected patients found that factors that influenced adherence to TB treatment positively were beliefs in the curability of TB, beliefs in the severity of TB in the presence of HIV infection and support from families and health professionals [16]. All patients of a qualitative study conducted in northern Ethiopia, reported the first two months of treatments were physically exhausting and geographical access to health care facilities and financial burdens were factors the most influenced timely TB treatment initiation and compliance [17].

Health care providers shared deep concern regarding risk of acquiring MDR- TB from patients while working as DOT provider in the TB clinics found in the three facilities included in this study. They felt their own vulnerability as first line service providers for TB and MDR-TB patients in the absence of standard TB infection control measures in the respective TB clinics. This could be due to the fact that all the three TB clinics in this study are also providing treatment services for MDR-TB patients. The health care providers suggested many strategies for improvement at the local level including implementing standard TB infection control measure in TB clinics, training of all health care providers on MDR-TB and design a motivation and recognition system for health care providers working TB and MDR-TB areas.

In this way, the study also indicated how facility based TB treatment supervision was very difficult to implement especially from patient’s point of view but if the system could be established the observation of TB treatment could have be done in different settings including at home not necessary by health care provider but also by HEWs and other TTSs for the whole six months of treatments and the national TB program in Ethiopia has started to decentralized TB care services beyond health facilities to make it more accessible to the community. TB treatment is being provided under observation by HEWs and TB treatment supporters in different settings including at home [4]. Related to this finding, a study conducted in southern Ethiopia has shown the involvement of HEWs in treatment observation at health posts improved treatment success rate [18] and this could be applied in different settings to ease the challenges and difficulties of patients in accessing health facility for DOT.

### Limitations of the study

In this study, TB treatment data was not reviewed. In this way, it was not possible to compare the patient and worker's lines. In addition, we did not observed the way that DOT is offered in the respective facilities.

## Conclusions

This study highlighted the several challenges and difficulties surrounding a facility based DOT as a method of TB treatment supervision in public health facilities in Addis Ababa. DOT was not being implemented for the full course of treatment particularly during the continuation phase of TB treatment as recommended by the National TB program (NTP) and this may have a risk for an increase in patients’ non compliance which is a major cause of development of MDR TB. The challenges and difficulties identified in this study may be indicative of situation in other Health facilities in Addis Ababa as well as elsewhere in the country.

Hence the NTP has to explore or do further researches on how best could the TB treatment delivery options improved to ensure adequate treatment adherences and minimize the negative implications of the daily burden imposed by the facility based DOT on TB patients. A more patient-centered approach could be introduced and strengthened by further decentralizing the DOT to the community level in order to ensure adherence of TB patients to their treatment. In addition, a system should be established to ensure the involvement of the community and family in TB prevention and control efforts as TB Treatment Supporters and the Health Care Providers concerns about the risk of acquiring MDR-TB while working in TB clinics should be further studied and addressed.

## Abbreviations

AACARHB: Addis Ababa City Administration Regional Health Bureau
AIDS: Acquired immunodeficiency syndrome
ART: Antiretroviral therapy
DOT: Directly observed therapy
DOTS: Directly observed treatment, short course
DP: DOT Provider
FDC: Fixed dose combinations
FGD: Focused group discussion
FMoH: Federal Ministry of Health
HBCs: High burden countries
HC: Health Center
HCP: Health care provider
HEW: Health extension worker
HIV: Human Immunodeficiency Virus
IC: Infection Control
IP: Interviewed patient
IRB: Institutional Review Board
MDR: TB Multiple Drug resistant TB
NTP: National TB program
OPD: Outpatient department
TB: Tuberculosis
TFC: Treatment follow-up center
TIC: Treatment Initiative Center
TSR: Treatment success rate
TTS: Tuberculosis treatment supporters
WHO: World Health Organization
